# End of the Beginning: Elongation and Termination Features of Alternative Modes of Chromosomal Replication Initiation in Bacteria

**DOI:** 10.1371/journal.pgen.1004909

**Published:** 2015-01-08

**Authors:** Jayaraman Gowrishankar

**Affiliations:** Laboratory of Bacterial Genetics, Centre for DNA Fingerprinting and Diagnostics, Hyderabad, India; A*STAR, Singapore

## Abstract

In bacterial cells, bidirectional replication of the circular chromosome is initiated from a single origin (*oriC*) and terminates in an antipodal terminus region such that movement of the pair of replication forks is largely codirectional with transcription. The terminus region is flanked by discrete *Ter* sequences that act as polar, or direction-dependent, arrest sites for fork progression. Alternative *oriC*-independent modes of replication initiation are possible, one of which is constitutive stable DNA replication (cSDR) from transcription-associated RNA–DNA hybrids or R-loops. Here, I discuss the distinctive attributes of fork progression and termination associated with different modes of bacterial replication initiation. Two hypothetical models are proposed: that head-on collisions between pairs of replication forks, which are a feature of replication termination in all kingdoms of life, provoke bilateral fork reversal reactions; and that cSDR is characterized by existence of distinct subpopulations in bacterial cultures and a widespread distribution of origins in the genome, each with a small firing potential. Since R-loops are known to exist in eukaryotic cells and to inflict genome damage in G1 phase, it is possible that cSDR-like events promote aberrant replication initiation even in eukaryotes.

## Introduction

Many features of chromosomal DNA replication are shared across the three kingdoms of life, including initiation from discrete origins, bidirectional fork progression, and termination by merger of opposing replication forks [1]. Whereas replication in eukaryotes is initiated from multiple origins on linear chromosomes, in bacteria most often there is a single circular chromosome whose replication is initiated from an *oriC* locus and proceeds bidirectionally for forks to meet in an antipodal terminus region. With this arrangement, replication and transcription of highly transcribed genes are rendered majorly codirectional in bacterial genomes. *oriC*-like sequences have been identified in more than 1,500 bacteria [2].

Alternative (*oriC*-independent) means of bacterial chromosomal replication have been characterized. These include (i) “integrative suppression” with replicons of phage or plasmids, and (ii) replication initiated from RNA–DNA hybrids or R-loops. The latter is called constitutive stable DNA replication (cSDR), whose mechanism is poorly understood. Significant perturbations, both of codirectionality between replication and transcription and of the arrangement for opposing replication forks to meet in the terminus region, are expected when bidirectional replication is not *oriC*-initiated.

This review explores the dynamics of fork progression and termination in *Escherichia coli* (gram-negative) bacterial cells exhibiting *oriC*-dependent and *oriC*-independent replication initiation to support two new concepts: (i) that when pairs of forks collide, bilateral fork reversal reactions take place; and (ii) that cSDR is characterized by stochastic replication initiation events distributed genome-wide. Table 1 summarizes relevant features and functions in *E. coli* that are shared in *Bacillus subtilis* (a gram-positive bacterium) and in eukaryotes, as is further elaborated in the text.

**Table 1:** Counterparts in *B. subtilis* and eukaryotes of *E. coli* functions related to chromosomal DNA replication and repair.

No.	*E. coli* features and functions (proteins) discussed in the text	Occurrence in	
		*B. subtilis*	Eukaryotes
1.	Bidirectional replication forks initiated from defined origin (single origin, *oriC*)	Similar to that in *E. coli* [4], [77], [112], [113]	Yes, but from multiple origins [1], [77], [96]
2.	Replication origin-binding protein essential for viability (DnaA)	Similar to that in *E. coli* [114], [115]	Yes (ORC proteins) [1], [77], [96]
3.	Essential replicative helicase in replisome (DnaB homohexamer, 5′-3′ polarity)	Yes (DnaC homohexamer, 5′-3′ polarity) [4], [112]	Yes (MCM2–7 heterohexamer in CMG complex, 3′-5′ polarity) [1], [96]
4.	Facilitation of replisome progression by accessory helicases (Rep, UvrD)	Yes (PcrA) [15], [116]–[118]	Yes (Rrm3) [12], [16]
5.	Fork disintegration and replication restart	Yes [119]–[121]	Yes [1], [9], [10], [96], [122]
6.	DNA repair by homologous recombination	Yes [121]	Yes [10], [121]–[126]
	a. Recombinase (RecA)	Similar to that in *E. coli* [121]	Yes (Rad51) [10], [121]–[125]
	b. Exonuclease resection at double strand ends (RecBCD)	Yes (AddAB) [122], [127]–[129]	Yes (MRX or MRN complex) [127], [129]–[131]
	c. Enzymes for Holliday junction migration and resolution (RuvABC)	Yes (RuvAB, RecU, RusA) [121], [132]	Yes (RAD54, GEN1, MUS81) [121]–[123]
7.	Replication fork reversal at stalled replisomes	Postulated, including during phage (SPP1) replication [15], [133], [134]	Yes [10]
8.	Completion of replication termination by merger of opposing replication forks	Similar to that in *E. coli* [12], [24], [25]	Yes [47]–[49], [135], [136]
9.	Polar arrest of replication fork progression at *Ter* sites (bound by Tus protein)	Similar to that in *E. coli* (*Ter* sites bound by Rtp protein) [12], [24], [25]	No
10.	Replication–transcription codirectionality in highly transcribed genes (such as rRNA genes)	Similar to that in *E. coli* [15], [120], [137], [138]	Yes [12], [13], [16], [139]
11	Rho-dependent termination of nascent untranslated (including antisense) transcripts	Yes [140]	No
12.	Transcription-associated R-loops	Not demonstrated	Yes [80]–[94], [141], [142]
	a. R-loop prevention by topoisomerase I action	Not demonstrated	Yes [85], [143]
	b. RNase H	Yes [144], [145]	Yes [146]
	c. RecG helicase	Similar to that in *E. coli* [132], [147], [148] (but see footnote *a* below)	Not demonstrated
13.	cSDR	Not demonstrated	Not demonstrated

*^a^*In an assay involving copy number determination of an R-loop dependent plasmid in *E. coli*, RecG from another gram-positive bacterium *Streptococcus pneumoniae* was shown to be active as an R-loop helicase but, unexpectedly, RecG from *B. subtilis* was inactive [147].

## Replication Initiated from *oriC* and Its Termination

**Figure 1:**
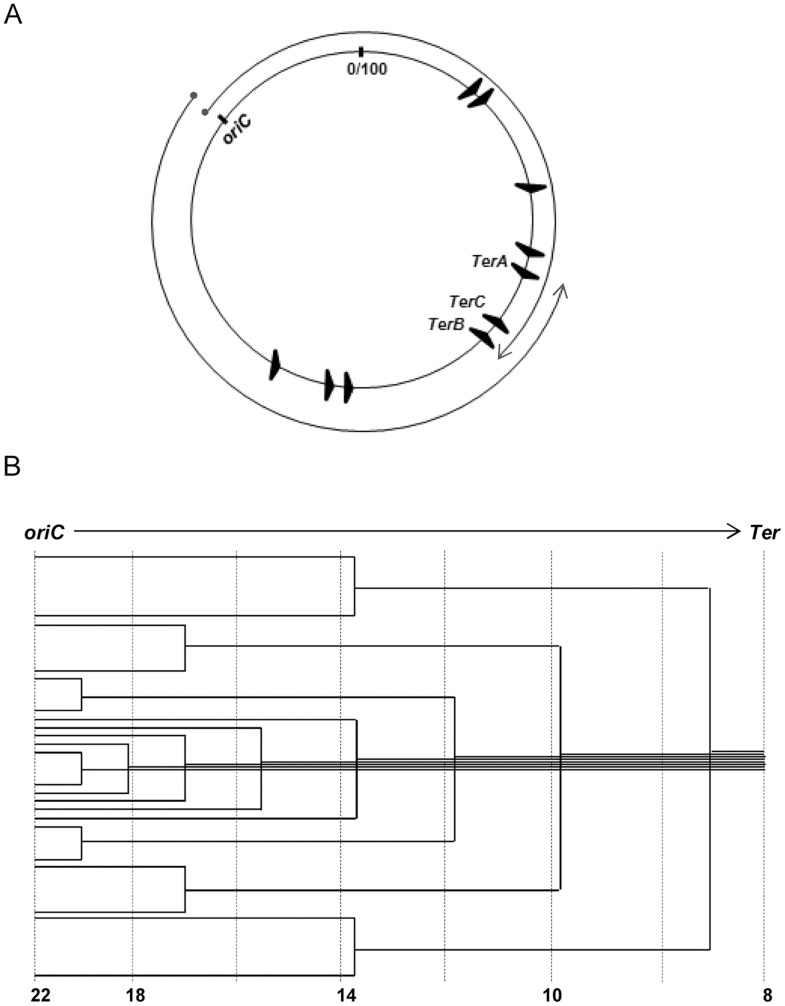
Features of *oriC*-initiated replication in *E. coli*. (**A**) Depiction of *oriC*, *TerA, TerB* and *TerC* loci on the 100 minute long circular *E. coli* chromosome, and of the clockwise and counterclockwise replichores; locations of the seven other *Ter* sites are also shown. (**B**) Schematic depiction of the copy number gradient, from *oriC* to *Ter*, created by the different extents to which replication forks have progressed on a single replichore in individual cells of an asynchronously dividing population. Aggregate copy numbers at the indicated positions are given at the bottom, but these are only illustrative and not to scale.

Bidirectional replication initiation at *oriC* is dependent on the protein DnaA, and included within the replisome complex at each fork is the 5′-3′ replicative helicase DnaB [3]–[6]. The forks move divergently around the circular chromosome to meet in the terminus region, and their traversed paths represent the (clockwise and counterclockwise) replichores (Fig. 1A). Both *oriC* and DnaA are essential for viability.

Advancing forks may suffer disintegration [7], [8], whose frequency is increased with DNA damage [7]–[10] or by transcription–replication conflicts [11]–[16]. For example, all seven ribosomal RNA operons are codirectional with the replichores, and inversion of any of them leads to slowing or disintegration of replication forks [17]–[19]. Accessory helicases Rep and UvrD with 3′-5′ polarity facilitate replisome progression across DNA–protein barriers, including at sites of transcription–replication conflict [15], [18]–[20]. Fork disintegration also occurs when one fork runs into a preceding one stalled on the same replichore [21], [22]. Reassembly of disintegrated forks is mediated by replication restart proteins acting together with the proteins for homologous recombination RecA, RecBCD, and RuvABC (Table 1) [7]–[10].

At the terminus region, the Tus protein binds to discrete *Ter* sequences and mediates polar, or direction-specific, arrest of replisome progression (by inactivating DnaB helicase) [12], [23]–[25]. Thus, this region contains at (i) its clockwise end, *TerA* where counterclockwise forks are terminated, and (ii) its counterclockwise end, *TerC* and *TerB* where clockwise forks are arrested (Fig. 1A). Hence, most often chromosomal replication is completed when opposing forks meet at, or in, the interval between *TerA* and *TerC* or *TerB*. However, replisome arrest at Tus-bound *Ter* sites is not absolute [26], [27]. In addition to *TerA, -B* and -*C*, seven other *Ter* sequences are present on the *E. coli* replichores (Fig. 1A), oriented such as to cause arrest only of the oppositely directed replisomes [12], [24], [28].

## Copy Number Analysis in Chromosome Replication Studies

When replication forks advance from origin to terminus in cells of an asynchronously dividing cell population, a decreasing gradient of gene copy numbers is expected from the former to the latter (Fig. 1B) [29], [30]. Analysis of copy number distributions has therefore enabled identification of origins and termini of replication [26], [27], [31]–[36] as well as of chromosome rearrangements [37]. Two caveats are (i) that copy numbers can change on account not only of fork progression but also of recombination (for example, tandem amplification [38]) or DNA degradation [39], [40]; and (ii) that the values represent an average of all cells in a population, which may comprise distinct subpopulations including inviable cells [38], [41].

## When Replisomes Collide: Evidence for Bilateral Replication Fork Reversals

Replication fork reversal is the process by which nascent leading and lagging daughter strands at a fork are extruded to anneal to one another, thus forming a cruciform or “chicken-foot” structure. Such extrusion could occur when replisome progression is halted for any reason, and would be promoted by accumulation of positive supercoils ahead of the fork. Fork reversals can competitively be either limited by “end-resection” activity of the RecBCD complex, or exacerbated by the RuvABC proteins that catalyze branch migration and cleavage at Holliday junctions [19], [42]–[45], reviewed in [10], [46]. In RecBC-deficient strains, fork reversal is also accompanied by excessive chromosome degradation (which may indeed seem paradoxical given that RecBCD is itself a potent DNA exonuclease), that is mediated by RecJ nuclease [19].

**Figure 2:**
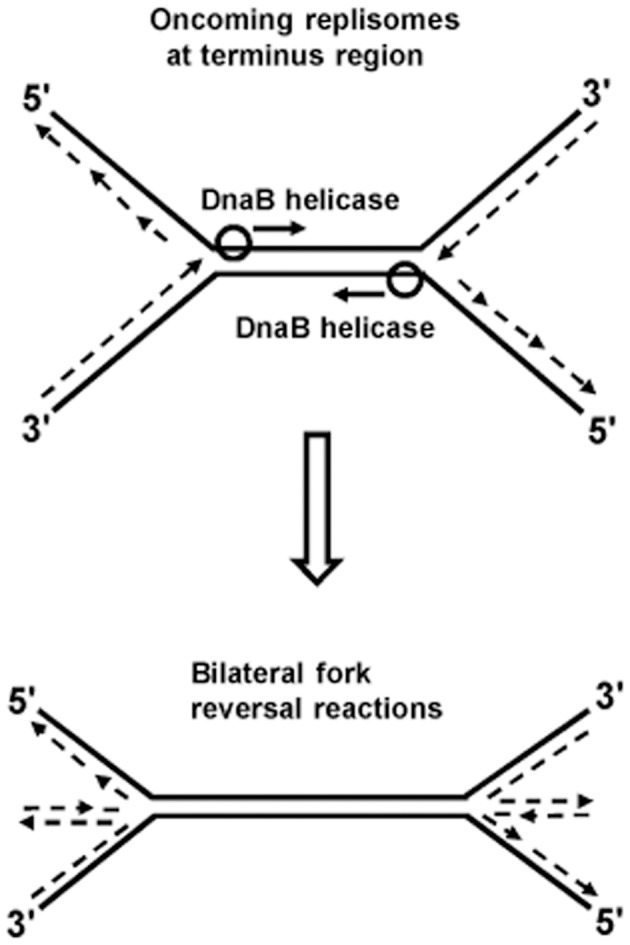
Model of bilateral fork reversal reaction at a site where oncoming replisomes meet during replication termination.

Kuong et al. [40] and Rudolph et al. [41] have shown that chromosomal terminus region copy numbers are severely reduced in RecBC-deficient strains (the former studies were done with thymine starvation). This suggests that irreparable chromosome breakage and degradation occurs in the terminus region of some proportion of *recBC* mutant cells which, for example, may result from bilateral fork reversals provoked by head-on collisions between two replisomes, as depicted in Fig. 2. Additional experiments are required to confirm this hypothesis. Since collisions between pairs of replisomes are a common feature of replication termination in all organisms [25], [47]–[49], it is also possible that consequential fork reversals may be universal.

## Chromosome Replication in Absence of *oriC* or DnaA: Integrative Suppression

In integrative suppression, the replication origin of a plasmid or phage is integrated into the chromosome of a strain defective in *oriC* or DnaA [26], [27], [33]–[36], [50]–[53]. In general, a strain's health is more compromised when the exogenous origin has integrated further from *oriC*, and when replication is unidirectional rather than bidirectional. Retrograde replication fork progression (towards *oriC*) in strains with ectopic origins is slow [27], [54], presumably because codirectionality between replication and transcription is lost, providing support to the model of impedance of fork movement by head-on transcription [17]–[19].

For strains where the exogenous origin is integrated at *oriC*-distal sites, the terminus region is replicated (as expected) by the fork which traverses the shorter arc between it and the integration site, but additionally there is a sharp decrease in copy numbers immediately before the *Ter* site that arrests its passage [26], [27]. A similar decrease in copy numbers proximal to the *Ter* arrest site of a prematurely arriving fork is evident in a strain possessing two chromosomal replication origins [41]. It is possible that these decreases are related to changes in fork architecture at the arrest sites, leading to DNA degradation by endo- and exonucleolytic enzymes.

## Chromosome Replication in Absence of *oriC* or DnaA: cSDR

Another means to confer viability to cells lacking *oriC* or DnaA is cSDR, wherein transcription-associated R-loops serve as primers for initial DNA synthesis following which replication forks are established by the mechanisms of replication restart [55], [56]. Enzymes RNase HI (*rnhA*-encoded) and RecG (*recG*-encoded) act to eliminate R-loops by hydrolysis and by unwinding, respectively, and DNA synthesis by cSDR has been demonstrated in both *rnhA* and *recG* single mutants (while the double-deficiency is lethal) [55]. R-loops similarly initiate replication in ColE1 plasmids [57], [58]. cSDR has also been implicated in stress-induced mutagenesis and genome instability [59].

## cSDR Origin Sites in RNase HI-Deficient Mutants

By examining the copy number gradient in *rnhA* mutants lacking *oriC*-initiated replication, the late Kogoma's group reported several putative replication initiation sites (termed *oriK*s), at least two of which were in the chromosomal terminus region [55]. Madiuke et al. [60] have revisited this question through a deep sequencing approach, and their results have again demonstrated a prominent copy number peak in the terminus region of *rnhA* mutants. However, this peak was abolished in a Tus-deficient derivative, leading the authors to suggest that it may not represent an *oriK* site but instead could be a consequence of trapping by polar *Ter* sites of replication forks that were initiated outside, and had then progressed into, the terminus region [60]. This idea is further developed in my model proposed below. Furthermore, no *oriK* locus was detected in a chromosome-wide search for sequences that could confer autonomous replication ability in an RNase HI-deficient strain [38], [61]. Thus, a definitive identification of the so-called *oriK* sites for cSDR has remained elusive.

## Where Do R-loops Occur in the *E. coli* Genome?

One way to identify replication initiation sites in cSDR would be to determine the locations of R-loops in the genome, even while it is recognized that their occurrence is necessary but may not be sufficient for establishing origin activity [62]. R-loop mapping studies have not been reported for *rnhA* or *recG* mutants, but they have been done [63] for a mutant defective in Rho-dependent transcription termination (RDTT) as explained below.

RDTT is a process by which nascent non-rRNA transcripts that are not being simultaneously translated are prematurely terminated. RDTT-deficient mutants exhibit an increased prevalence of R-loops [63]–[65], which is assumed to arise from the reannealing of nascent untranslated transcripts with upstream DNA [66], [67]. R-loop formation in these situations is facilitated also by backtracking of RNA polymerase leading to stalled or arrested transcription elongation complexes [68], [69], but why this is so is unclear.

In an RDTT-deficient mutant, R-loops are distributed genome-wide, being generated from both sense and antisense transcripts [63]; Peters et al. [70] have also shown that antisense transcription is increased when RDTT is compromised (reviewed in [71]). Therefore, it is likely that *oriK* sites for cSDR are also widespread, and that they may indeed be stochastically different amongst individual cells in a population. This would explain the earlier findings [60], [61] that no distinct *oriK* sites were unambiguously identified in RNase HI-deficient mutants.

## cSDR in RecG-Deficient Mutants

### cSDR with RNase HI- or RecG-deficiency: Similar findings, different models

Copy number studies in both *rnhA* [60] and *recG* [41] mutants have demonstrated the similar occurrence in them of Tus-dependent (i) peak in the chromosomal terminus region, and (ii) inversion of the classical *oriC*-peaked curve when replication initiation from *oriC* is abolished. However, cSDR in the *recG* mutant has been explained to be the consequence of aberrant replication reinitiation events following fork collisions [41], [72].

According to this model [41], when opposing forks meet in the terminus region, DnaB helicase acts to unwind and extrude the oncoming fork's leading strand at its 3′ end, which then serves as a substrate for aberrant replication restart unless RecG and at least one of three single-strand DNA 3′ exonucleases are available. Combined deficiency of RecG together with the three 3′ exonucleases is lethal [73], which has been attributed to excessive occurrence of such over-replication in these cells. However, the source of origin of forks which are postulated to collide in the terminus region to mediate cSDR in *recG* mutants has not been explained, since these strains were also DnaA-deficient [41].

This raises the question of replication initiation in cSDR occurring by completely different mechanisms in *rnhA* and *recG* mutants, the former from R-loops [55], [60] and the latter from fork collisions in the terminus region [41]. However, the similarities cited above would suggest that cSDR in both indeed operates by a common mechanism, as is further explored below. An additional similarity is that, just as with RecG deficiency, RNase HI deficiency is also lethal in the combined absence of the three 3′ exonucleases [73].

### A model invoking subpopulations with distinct replication dynamics during cSDR

**Figure 3:**
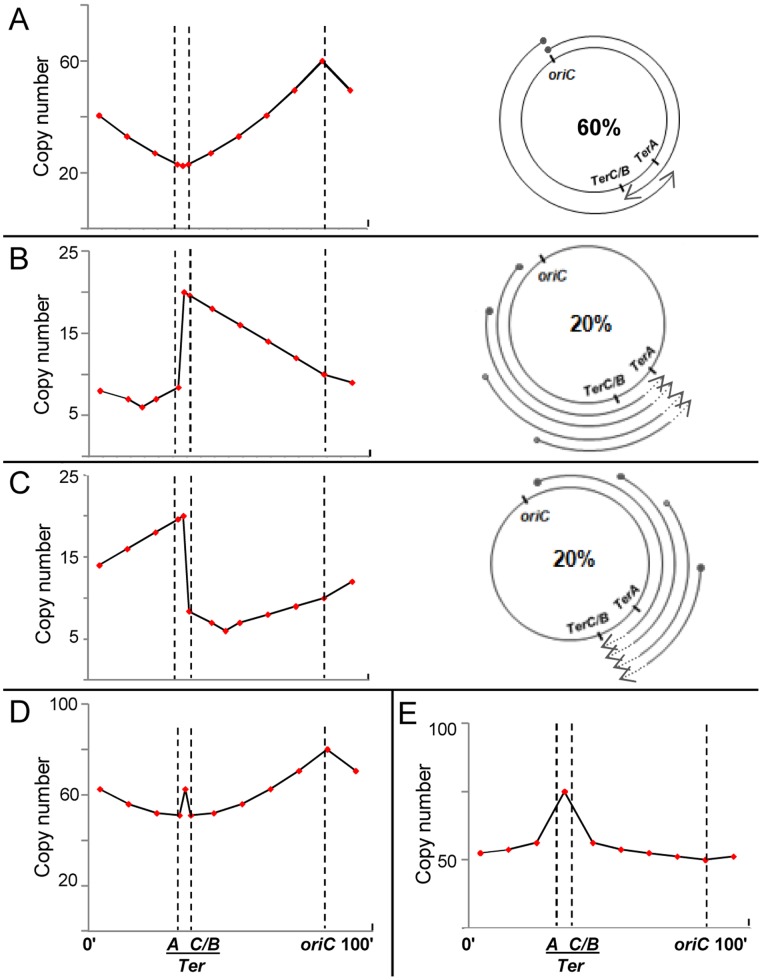
Predicted copy number distribution patterns for different categories of replication events in *recG* or *rnhA* mutants. For all curves, positions of *oriC*, *TerA*, and *TerC* or *TerB* (*TerC/B*), are marked by the interrupted vertical lines; and copy number values are plotted on a linear instead of log scale to enable comparison with curves shown in Rudolph et al. [41]. (A–C) Three categories of replication events are shown, comprising those with forks initiated, respectively, (i) at *oriC*, DnaA-mediated (60%); (ii) on the counterclockwise replichore at various locations, R-loop mediated (20%); and (iii) on the clockwise replichore at various locations, R-loop mediated (20%). An individual cell in the population may harbor more than one category of event (see text). On the right in each of the three panels is a schematic depiction of progression of forks, each beginning at a solid circle and progressing to the position of arrowhead; in panels B and C, terminus region chromosomal DNA degradation (proximal to the sites of fork arrest at *Ter*) is shown as interrupted lines on the arcs, but retrograde fork advancements towards *oriC* (which are expected to occur at low efficiency [27], [54]) are not marked. On the left in each of the three panels is shown the expected copy number distribution for that category. (D) Expected copy number distribution for the entire cell population, obtained by summation of the distributions shown in panels A–C. (E) Expected copy number distribution for *recG* or *rnhA* mutant lacking *oriC*-initiated replication.

The sharpness of the observed copy number peak in the terminus region of *recG* mutants is inconsistent with the excessive reinitiation model [41], which would predict that copy numbers exhibit a plateau (with no peaks) across this entire interval between *TerA* and *TerB* or *TerC* (since replication reinitiation anywhere within this region will duplicate all markers between the Tus-bound *Ter* boundaries). An alternative way to explain the observed peak in the mid-terminus region of a *recG* or *rnhA* mutant is to assume that its copy number curve is a composite of distributions from subpopulations with one or more of three different kinds of replication initiation events, as represented in Fig. 3.

For the purpose of this depiction, 60% of replication initiations are envisaged to have occurred from *oriC* (Fig. 3A), and 20% each from R-loops in the counterclockwise and clockwise replichore arms (Figs. 3B and 3C, respectively). Nevertheless, a single cell may harbor more than one category of replication event: for example, a simple representation of the percentages above would have it that for every three cells in the culture per generation, one suffers a (supernumerary) cSDR initiation event on the clockwise replichore, another a similar event on the counterclockwise replichore, whereas all exhibit *oriC*-initiated replication.

For cSDR events initiated from sites on the counterclockwise replichore (Fig. 3B), retrograde progression of forks towards *oriC* would be slow (as noted in other cases earlier [27], [54]), whereas they would progress smoothly towards and beyond *TerC/B* into the terminus region to be arrested at *TerA*; the small proportion of forks that overcome arrest would then progress in retrograde direction beyond *TerA*. Since R-loops are evenly distributed [63], cSDR origins are also likely to occur at a uniform, but low, probability across the genome, such that the copy number for an arbitrary locus on the counterclockwise arm is higher the further its distance from *oriC* (which is opposite to that with DnaA-mediated initiations from *oriC*; compare Figs. 3A and 3B).

The earlier studies [26], [27], [41] have also indicated that prolonged arrest of replication forks at a *Ter* site, in the absence of arrival of forks of the opposite replichore, is associated with a sharp copy number drop in the region preceding the fork arrest site (which is depicted in Figure 3B adjacent to *TerA*, in the interval between *TerA* and *TerC* or *TerB*). The mirror symmetrically reverse situation would apply for cSDR initiation events on the clockwise replichore, as shown in Figure 3C.

The composite pattern for the entire population, derived by summation of the three distributions above, is shown in Figure 3D. Two features of the data reported for mutants lacking RecG [41] or RNase HI [60] are recapitulated here, namely, a peak in the mid-terminus region and a smaller enrichment of *oriC*-proximal to *oriC*-distal markers (compare Figs. 3A and 3D). As has also been suggested earlier [41], many cSDR events may likely contribute only to copy number values without concomitantly increasing viable cell numbers, since every event would not necessarily lead to duplication of the entire chromosome.

With the same assumptions, the copy number distribution in an *rnhA* or *recG* mutant that is additionally defective for DnaA can be derived as the approximate composite of Figs. 3B and 3C, as depicted in Fig. 3E. The derived curve broadly recapitulates the inversion in these mutants of the “classical” curve so that the peak and trough are now at the terminus and *oriC*, respectively [41], [60].

In strains exhibiting cSDR, the frequency of replication fork disintegration events is expected to be elevated when replisomes advance towards *oriC*; this would explain their dependence for viability on proteins of replication restart and homologous recombination [55], [56]. Since R-loop prevalence is less upon loss of RecG than of RNase HI [55], cSDR-mediated viability of *a recG dnaA* mutant requires the presence of additional mutations in Tus and RNA polymerase (rpoB*35) [41]. While absence of Tus permits passage of counterdirectional forks across *Ter* sites, rpoB^*^35 mitigates the adversity associated with transcription–replication conflicts [44], [68], [74]; in cSDR, rpoB^*^35 would promote retrograde fork progression both from cSDR initiation sites and in regions beyond the *Ter* sites.

## Comparisons in Other Organisms

The similarities listed in Table 1 between *E. coli* and *B. subtilis* would suggest that the models proposed here for the former may apply to the latter, although cSDR has so far not been demonstrated in *B. subtilis*. Archaeal and eukaryotic DNA replication mechanisms are very similar [1], [75]–[77], and in the archaeon *Haloferax volcanii,* the circular chromosome possesses four replication origins, but derivatives in which all were deleted unexpectedly exhibited greater fitness than the parental strains [78]; a cSDR-like mechanism has been proposed in the originless mutant [78], although an alternative possibility is that dormant replication origins were activated under these conditions [79].

Transcription-associated R-loops exist in eukaryotes [80]–[84], and their prevalence is increased when either elongation or cotranscriptional processing of mRNA is impeded [85]–[94]. DNA double-strand breaks occur in the G1 phase following R-loop formation [81], but the mechanism is not known, and one could thus speculate whether cSDR-like events may be a contributory factor. Furthermore, replication stress in eukaryotes triggers new initiation sites that are generally thought to arise by activation of dormant origins [1], [10], [95]–[98]; once again, it is possible that some of them arise from R-loops, given that they are located predominantly in transcribed gene regions [99], [100]. That R-loops in eukaryotic cells may confer genome instability by priming new DNA synthesis has been suggested earlier [59], [86]–[88], [101]; the BRCA2 protein, which functions as “chromosome custodian” and cancer suppressor [102], has also recently been suggested to exert its oncoprotective role by preventing R-loop accumulation [84].

## Conclusions and the Future Perspective

The major ideas proposed in this review, which need to be tested in future studies, are that fork reversal reactions occur when opposing replisomes meet; that replication origins for bacterial cSDR are widespread in the genome (although the firing potential of any single origin is small); that replication fork progression in cSDR faces two separate obstacles, of conflicts with transcription and of arrest at Tus-bound *Ter* sites; and that cSDR-like events may contribute to R-loop mediated genome damage in eukaryotes.

The additional questions to be addressed in the bacterial systems include the following [67]: What are the determinants of R-loop propensity? What regulates conversion of R-loops to replication origins? Would cSDR occur in other instances of increased R-loop prevalence, such as in mutants deficient in Rho (discussed above) or topoisomerase I [103]–[105]? When forks undergo polar arrest at Tus-bound *Ter* sites in absence of oncoming forks, how does the postulated DNA degradation occur proximal to *Ter*? And what are the roles for single-strand DNA exonucleases in replication?

The bacterial chromosome is organized into macrodomains [106]–[109], one of which is the Terminus macrodomain. Whether such organization influences (or is influenced by) replication fork dynamics near the terminus is unclear. Chromosome replication is also linked to downstream events of chromosome segregation, nucleoid condensation and cytokinesis [5], [110], [111], and the repercussions thereon of perturbations in replisome progression remain to be characterized.
